# Norbin Stimulates the Catalytic Activity and Plasma Membrane Localization of the Guanine-Nucleotide Exchange Factor P-Rex1*[Fn FN2]

**DOI:** 10.1074/jbc.M115.686592

**Published:** 2016-01-20

**Authors:** Dingxin Pan, Mark A. Barber, Kirsti Hornigold, Martin J. Baker, Judit M. Toth, David Oxley, Heidi C. E. Welch

**Affiliations:** From the ‡ Signalling Programme and; the § Mass Spectrometry Facility, Babraham Institute, Babraham Research Campus, Cambridge CB22 3AT, United Kingdom

**Keywords:** cell signaling, G protein-coupled receptor (GPCR), guanine nucleotide exchange factor (GEF), protein complex, Rac (Rac GTPase), Rho (Rho GTPase), small GTPase, Neurochondrin (NCDN), PREX1, PREX2

## Abstract

P-Rex1 is a guanine-nucleotide exchange factor (GEF) that activates the small G protein (GTPase) Rac1 to control Rac1-dependent cytoskeletal dynamics, and thus cell morphology. Three mechanisms of P-Rex1 regulation are currently known: (i) binding of the phosphoinositide second messenger PIP_3_, (ii) binding of the Gβγ subunits of heterotrimeric G proteins, and (iii) phosphorylation of various serine residues. Using recombinant P-Rex1 protein to search for new binding partners, we isolated the G-protein-coupled receptor (GPCR)-adaptor protein Norbin (Neurochondrin, NCDN) from mouse brain fractions. Coimmunoprecipitation confirmed the interaction between overexpressed P-Rex1 and Norbin in COS-7 cells, as well as between endogenous P-Rex1 and Norbin in HEK-293 cells. Binding assays with purified recombinant proteins showed that their interaction is direct, and mutational analysis revealed that the pleckstrin homology domain of P-Rex1 is required. Rac-GEF activity assays with purified recombinant proteins showed that direct interaction with Norbin increases the basal, PIP_3_- and Gβγ-stimulated Rac-GEF activity of P-Rex1. Pak-CRIB pulldown assays demonstrated that Norbin promotes the P-Rex1-mediated activation of endogenous Rac1 upon stimulation of HEK-293 cells with lysophosphatidic acid. Finally, immunofluorescence microscopy and subcellular fractionation showed that coexpression of P-Rex1 and Norbin induces a robust translocation of both proteins from the cytosol to the plasma membrane, as well as promoting cell spreading, lamellipodia formation, and membrane ruffling, cell morphologies generated by active Rac1. In summary, we have identified a novel mechanism of P-Rex1 regulation through the GPCR-adaptor protein Norbin, a direct P-Rex1 interacting protein that promotes the Rac-GEF activity and membrane localization of P-Rex1.

## Introduction

P-Rex1 is a guanine nucleotide exchange factor (GEF)^3^ that activates the Rac-type small G proteins Rac1, Rac2, Rac3, and RhoG; a branch of the Rho-family of GTPases (1, 2). P-Rex1 is highly expressed in leukocytes and brain, but found at lower levels in many other cell types and tissues (2). By activating Rac, P-Rex1 controls a wide range of cell responses, including processes dependent on actomyosin cytoskeletal dynamics such as changes in cell morphology, adhesion, and chemotaxis, but also reactive oxygen species production and gene expression (2). Through these processes, P-Rex1 is important in the proinflammatory functions of leukocytes and platelets (1–9), in neuronal morphology and synaptic plasticity (10–12), and in the proliferation and migration of melanocytes during development (13–15). Deregulation of the *PREX1* gene is common in several types of human cancers, including melanoma, breast and prostate cancer, with overexpressed P-Rex1 promoting tumor growth and/or metastasis (2, 13, 16, 17).

P-Rex1 is comprised of a catalytic DH domain in tandem with a PH domain, as is typical for Dbl family Rac-GEFs, two pairs of PDZ and DEP domains, and a C-terminal half that shares weak homology with inositol polyphosphate 4-phosphatase (IP4P) (1, 2). The Rac-GEF activity of P-Rex1 is known to be regulated by three mechanisms. It is directly stimulated by phosphatidylinositol (3,4,5)-triphosphate (PIP_3_), the lipid second messenger produced by phosphoinositide-3 kinase (PI3K), and by the Gβγ subunits of heterotrimeric G proteins that are released upon activation of G-protein-coupled receptors (GPCRs), and it is also modulated by serine phosphorylation (1, 2). PIP_3_ and Gβγ stimulate P-Rex1 GEF activity robustly both independently and in synergy (1), with PIP_3_ binding to the PH domain and Gβγ binding to the DH domain being sufficient *in vitro* (18, 19). Molecular modeling based on a recent crystal structure suggested that the Gβγs dock on the opposite face of the DH domain than the Rac1-binding site, and that they also contact the PH domain (20). However, in the cell, additional P-Rex1 domains contribute to the activation by Gβγ (21). In addition, P-Rex1 activity can be directly stimulated by the protein phosphatase PP1α through dephosphorylation of Ser^1165^ (22) and inhibited by the PKA through phosphorylation of unidentified sites (23). In breast cancer cells, P-Rex1 can also be activated upon phosphorylation of Ser^1169^, by unidentified serine kinases, in response to cell stimulation through receptor tyrosine kinases (24, 25). Finally, as well as stimulating the Rac-GEF activity of P-Rex1, PIP_3_ and Gβγ also control the subcellular localization of P-Rex1, by synergistically promoting its plasma membrane localization, thus bringing the GEF, which is mainly cytosolic under basal condition, into close proximity with its substrate GTPase Rac (26, 27).

Complex formation with other proteins is a common mechanism of GEF regulation (28). However, few binding partners of P-Rex1 have been identified to date (2). Apart from its substrate Rac and the regulators mentioned above, P-Rex1 has been shown to interact directly with the mammalian target of rapamycin complexes TORC1 and TORC2 through its DEP domains, but the functional consequences for both P-Rex1 and mammalian target of rapamycin signaling remain unclear (29). Furthermore, the P-Rex1 homologue P-Rex2, but not P-Rex1 itself, interacts directly with the tumor suppressor PTEN, leading to the inhibition of both the PTEN phosphatase and P-Rex2 Rac-GEF activities (30–32).

To search for potential new regulators of P-Rex1, we therefore carried out a screen for P-Rex-binding proteins, and thus identified Norbin, also known as Neurochondrin (NCDN). Norbin is a 79-kDa cytosolic protein that is highly conserved throughout vertebrates, harbors no catalytic activity or homologies with other proteins or domains, is predicted to have armadillo repeat structure, and is abundantly expressed in the nervous system, bone and cartilage tissue (33–37). Norbin was originally identified as a promoter of neurite outgrowth (33, 38) and of the bone resorptive function of osteoclast-like bone marrow cells (35). General Norbin deficiency in the mouse is early embryonic lethal (39), but targeted deletion in the nervous system (40) or specifically the postnatal forebrain (41, 42), causes defects in spatial learning and synaptic plasticity, leading to depression- and schizophrenia-like behaviors.

Norbin binds as an adaptor to the cytoplasmic C termini of many GPCRs, 33 of 45 GPCRs from different classes investigated to date (41, 43, 44). Norbin binding to these GPCRs was shown to be direct and to modulate the activity and/or the trafficking of the receptor in a manner that depends on the type of GPCR. Whereas coexpression of Norbin together with the GPCRs for melanin-concentrating hormone, thromboxane, or orexin in HEK-293 cells attenuated ligand-stimulated intracellular calcium rises (43, 44), coexpression of Norbin with the glutamate receptor mGluR5 increased phospholipase C activity, calcium rises, and ERK activity in a ligand-dependent manner (41). Similarly, whereas Norbin expression did not affect the ligand-induced internalization of melanin-concentrating hormone receptor 1 in HEK-293 cells (44), it did control the steady-state cell surface levels of mGluR5 in Neuro-2a cells and mouse brain cortical neurons (41). In addition to GPCRs, Norbin was reported to bind the signaling phospholipid phosphatidic acid (45), the transmembrane protein semaphorin 4C (46), and the Rho-GTPase effector Dia1 (47) *in vivo*, although the functional consequences of these interactions remain unknown.

In this paper, we report the identification of Norbin as a new direct binding partner of P-Rex1. We characterize the effects of Norbin on the Rac-GEF activity and subcellular localization of P-Rex1, and on the Rac1-dependent role of P-Rex1 as a regulator of cell morphology.

## Experimental Procedures

### 

#### 

##### Isolation of Norbin from Mouse Brain Cytosol Fractions with Recombinant EE-P-Rex1

The brains of 20 P-Rex1^−/−^ mice (6) were removed immediately after sacrifice, snap-frozen in liquid nitrogen, wrapped in cling-film, and the frozen tissue dissociated with a hammer on a metal tray on dry ice. The homogenized tissue (8 g) was resuspended in 40 ml of ice-cold, detergent-free brain tissue resuspension buffer (150 mm NaCl, 20 mm HEPES (pH 7.2 at 4 °C), 1 mm EDTA, 5 mm EGTA, 1 mm DTT, 20 mm β-glycerophosphate, 25 mm NaF, 1 mm Na_3_VO_4_ (pH 10.2), 0.1 mm PMSF, 10 μg/ml each of leupeptin, pepstatin A, aprotinin, and antipain), and cells were lysed by sonication on ice with 20 × 1-s on/off pulses using the large tip of a Misonix 3000 probe sonicator. The lysate was cleared of insoluble material by ultracentrifugation at 150,000 × *g* for 90 min at 4 °C, and Tween 20 was added to the 45 ml of supernatant (cytosol fraction) to a concentration of 0.03%. Salt was removed by loading the cytosol onto a PD10 desalting column (5 × 14 cm Sephadex G-25M, Amersham Biosciences) that had been equilibrated in 20 volumes of column buffer 1 (30 mm Tris-HCl, pH 8 at 4 °C, 1% betaine, 0.1 mm EDTA, 0.1 mm EGTA, 0.1 mm Na_3_VO_4_, and 0.01% sodium azide) at a flow rate of 2 ml/min and by washing with 100 ml of ice-cold column buffer 2 (column buffer 1 containing 0.03% Tween 20, 10 mm DTT, 1 mm PMSF, and 250 μg/ml each of leupeptin, pepstatin-A, aprotinin, and antipain) at the same flow rate. A single 55-ml fraction containing the total desalted cytosolic protein (9 mg/ml) was recovered and loaded onto a 2× 60-cm Source 15Q anion exchange column that had been equilibrated with ice-cold column buffer 3 (column buffer 1 containing 0.03% Tween 20, 1 mm DTT, 0.1 mm PMSF, and 10 μg/ml each of leupeptin, pepstatin-A, aprotinin, and antipain) at 2 ml/min. A continuous gradient of up to 0.5 m NaCl was applied over 400 ml by mixing in column buffer 4 (column buffer 1 containing 1 m NaCl, 1 mm DTT, 0.1 mm PMSF, and 10 μg/ml each of leupeptin, pepstatin-A, aprotinin, and antipain), with a final elution at 1 m NaCl in column buffer 4. Fifteen fractions were collected, ranging from the flow-through to the 1 m NaCl fraction and from 10 to 50 ml in volume, depending on the protein profile. The salt concentration of each fraction was adjusted to 150 mm by the addition of 3 m NaCl or by dilution with column buffer 3, as appropriate, and stored at 4 °C overnight. The next day, fractions were pre-cleared for 1 h with 100 μl of Sepharose CL-4B (Sigma) to minimize nonspecific protein binding to beads later on. Each cytosol fraction was then split in two, and one-half incubated with 10 μg of purified recombinant human full-length EE-P Rex1 protein that had been immobilized using EE antibody covalently coupled to protein G-Sepharose (48), the other half with EE-antibody beads alone (without EE-P-Rex1), as a control. After a 2-h incubation at 4 °C with end-over-end rotation, beads were loaded into microspin® columns (Pierce) and washed 3 times with column buffer 2. Protein was denatured in boiling Laemmli sample buffer, resolved on 8% SDS-PAGE gels, and stained using the SilverQuest^TM^ silver staining kit (Invitrogen). Protein bands seen in the presence but not absence of EE-P-Rex1 in corresponding fractions were excised, subjected to various proteolytic digests, and identified by liquid chromatography mass spectrometry (LC/MS) using an Applied Biosystems/MDS SCIEX Q-Star Pulsar i quadrupole time-of-flight tandem mass spectrometer coupled to a nanoLC system, with operation in nano-electrospray mode. Data were evaluated against eukaryotic entries in Uniprot 13.4, and obvious contaminants (trypsin, keratin, P-Rex1 fragments) were excluded.

##### Western Blotting

Tissue lysates from adult P-Rex1^+/+^ and P-Rex1^−/−^ mice were prepared as previously described (6, 10). Proteins from these and other lysates, prepared as described below, were denatured in boiling Laemmli sample buffer, resolved by SDS-PAGE, and transferred onto PVDF by wet transfer. Membranes were blocked in TBS, 0.05% Tween 20 containing 5% nonfat milk or 1% (w/v) BSA, as appropriate. Primary antibodies were used as previously described: myc and EE mouse mABs (Babraham Monoclonal Antibody Unit) (1, 10, 26), P-Rex1 mouse mAB 6F12 (from Prof Marcus Thelen, Institute for Research in Biomedicine, Bellinzona, Switzerland) (6), rabbit pABs Norbin C1, C3, N4, and N7 (from Dr. Kei Maruyama, Tokyo Institute of Psychiatry, Japan) (33), GST and β-actin mouse mABs (Sigma), HRP-coupled myc-AB P/N 46-0709 (Invitrogen), and Rac1 mouse mAB (Millipore 05-389) (6). Secondary antibodies used were HRP-conjugated goat anti-mouse IgG (Bio-Rad) or goat anti-rabbit IgG (Santa Cruz). Detection was done with Amersham Biosciences ECL or ECL-Plus and Kodak x-ray film. Where required, membranes were stripped in 25 mm glycine (pH 2.0), 1% SDS and reprobed. For densitometric analysis, films were scanned and band intensities quantified using ImageJ. To test total protein loading, PVDF membranes were stained with Coomassie after blotting.

##### Expression Constructs

Full-length human P-Rex1 cDNA constructs in pCMV3(EE), pCMV3(myc), and pEGFP vectors for the expression of N terminally epitope-tagged proteins in mammalian cells were described previously (1, 18, 22, 48). Full-length human Norbin in pCMV3(myc) vector (45) was a gift from Dr. Nicholas Ktistakis, Babraham Institute, UK. Deletion and truncation mutants of P-Rex1, all as described previously (18, 48), were subcloned from pAcoG1 into pCMV3(EE) and pEGFP vectors using appropriate restriction enzyme sites, and constructs were verified by sequencing.

##### Cell Culture and Transfection

Mammalian cell lines were used between 1 and 12 weeks in culture at 37 °C in a humidified incubator with 6% CO_2_ (or 5% CO_2_ for COS-7 cells) in Nunc tissue-culture flasks and passaged by trypsinization every second day. Human embryonic kidney (HEK-293) and COS-7 cells were maintained in Dulbecco's modified Eagle's medium (DMEM, Gibco), 10% fetal bovine serum (FBS), 100 IU/ml of penicillin, and 100 μg/ml of streptomycin. Porcine aortic endothelial (PAE) cells that stably express the PDGFβ receptor were cultured in Ham's F-12 (Gibco), 10% FBS, 100 IU/ml of penicillin, and 100 μg/ml of streptomycin. COS-7 cells were transfected by electroporation as previously described (22). HEK-293 or PAE cells were transfected 1 day after seeding in complete medium at 1 × 10^5^ to 1.2 × 10^5^ cells/ml on Nunc plastic or 22-mm sterile glass coverslips, as appropriate, using JetPEI (Polyplus) according to the manufacturer's instructions. For down-regulation of endogenous Norbin, HEK-293 cells were transfected with Norbin siRNA1, AGACCUCAUCCUUGCGUAA (Dharmacon, UK, J-012835-19-0005), Norbin siRNA2, AGGCCAAGAAUGACAGCGA (J-012835-20-0005), or a pool of four non-targeting siRNAs (D-001810-10) as a control, using Lipofectamine (Life Technologies, UK) according to the manufacturer's protocol. Briefly, HEK-293 cells in DMEM, 10% FBS (without antibiotics) were cultured for 5 h with siRNA/Lipofectamine and a further 48 h in complete medium. Total lysates were prepared by scraping HEK-293 cells into ice-cold cell lysis buffer (50 mm Tris, pH 7.4, 10% glycerol, 100 mm NaCl, 1% Nonidet P-40, 2 mm MgCl_2_, 1× Complete Protease Inhibitor Mixture^TM^ (Roche Applied Science)), incubation on ice for 5 min with repeated vortexing, and removal of insoluble material at 16,000 × *g* for 10 min at 4 °C prior to denaturing in boiling Laemmli sample buffer.

##### Coimmunoprecipitation of EE-P-Rex1 and myc-Norbin from COS-7 Cells

COS-7 cells were transfected to express EE-P-Rex1 and/or myc-Norbin, or were mock-transfected, as described above and grown in DMEM, 10% FBS for 16 h and in DMEM, 10% FBS, 100 IU/ml of penicillin, and 100 μg/ml of streptomycin for another 24 h. Cells were collected by trypsinization, washed twice in ice-cold PBS, and resuspended in 1.3 ml of COS-7 resuspension buffer (20 mm HEPES, pH 7.2 at 4 °C, 150 mm NaCl, 1 mm EDTA, 5 mm EGTA, 0.03% Tween 20, 1 mm DTT, 0.1 mm PMSF, and 10 μg/ml each of leupeptin, pepstatin-A, aprotinin, and antipain). Cells were lysed by probe sonication using the microprobe of an MSE Soniprep 150 sonicator (Sanyo) with 3 × 15-s on/off pulses on ice, followed by the addition of 20% Triton X-100 in PBS to a final concentration of 1%, with incubation for 10 min on ice. For experiments comparing full-length and mutant EE-P-Rex1, cells were lysed in RIPA buffer (30 mm HEPES, pH 7.4 at 4 °C, 150 mm NaCl, 1% Nonidet P-40, 0.5% sodium deoxycholate, 0.1% SDS, 5 mm EGTA, 4 mm EDTA, 0.1 mm PMSF, and 10 μg/ml each of leupeptin, pepstatin-A, aprotinin, and antipain) for 10 min on ice. The soluble fraction was prepared by ultracentrifugation at 200,000 × *g* for 60 min at 4 °C. A 50-μl aliquot was retained as a total lysate control for Western blotting and boiled in Laemmli sample buffer. The remainder was incubated with EE antibody covalently coupled to protein G-Sepharose for 60 min at 4 °C with end-over-end rotation. Beads were washed 4 times in COS-7 resuspension buffer containing 1% Triton X-100 (or in RIPA buffer for assays with mutants) and boiled in Laemmli sample buffer. Coimmunoprecipitation and total lysate samples were subjected to SDS-PAGE and Western blotting with myc, EE, or HRP-coupled myc antibodies.

##### Coimmunoprecipitation of Endogenous P-Rex1 and Endogenous Norbin from HEK-293 Cells

Eight 175-cm^2^ tissue culture flasks containing HEK-293 cells grown to 80–90% confluence in complete medium were washed twice in PBS and placed on an ice-cold metal tray. Cells were scraped into 9 ml of ice-cold cell lysis buffer and incubated on ice for 10 min with occasional vortexing. Lysates were spun at 1,500 × *g* for 10 min at 4 °C to remove cell debris, and the supernatant was ultracentrifuged at 100,000 × *g* for 30 min at 4 °C. The supernatant (soluble fraction) was pre-cleared with 600 μl of Sepharose CL-4B, before being divided into two 4-ml aliquots. The remainder was retained as a total lysate control for Western blotting. One 4-ml aliquot was incubated with a pool of three Norbin antibodies (C1, N4, and N7) (33) for 90 min at 4 °C with end-over-end rotation, the other mock-treated with 20 μg of human serum IgG (Sigma). Twenty-five microliters of protein A-Sepharose were added for a further 45 min, beads were washed 3 times in cell lysis buffer, and proteins were denatured in boiling Laemmli sample buffer, resolved by SDS-PAGE, and Western blotted with Norbin C3 (33) or P-Rex1 6F12 antibodies (6).

##### Production and Purification of Recombinant Proteins

Human recombinant full-length (1) and mutant EE-P-Rex1 proteins (18), and prenylated Gβ_1_γ_2_ proteins were produced by baculovirus-driven expression in Sf9 insect cells and purified using their EE-tags, as previously described (48). For the production of GST-Norbin and GST proteins, competent *Escherichia coli* BL21(DE3) pLysS (Stratagene) were transformed with pGEX-Norbin or pGEX vectors, and protein expression was induced by the addition of 0.5 mm isopropyl 1-thio-β-d-galactopyranoside to 400 ml of exponentially growing cultures for 2.5 h. Bacteria were sedimented at 1500 × *g* for 20 min at 4 °C, resuspended in 20 ml of ice-cold PBS, 2 mm EGTA, 1× mini protease inhibitor mixture (Roche), 0.1 mm PMSF, and 0.1 mm DTT, and were sonicated using the large probe of an MSE Soniprep 150 sonicator (Sanyo) with 5 pulses of 15 s on/off on ice. Triton X-100 was added to 1% (v/v) from a 20% stock in PBS and the lysate incubated for 40 min on ice with occasional mixing. Insoluble material was removed by ultracentrifugation at 100,000 × *g* for 1 h at 4 °C, and the supernatant was incubated with 500 μl of pre-washed glutathione-Sepharose 4B for 2 h at 4 °C with end-to-end rotation. Beads were washed 3 times in PBS, 1% Triton X-100, 1× mini-proteinase inhibitor tablets (Roche), and stored at 4 °C for up to 1 week. To estimate the amounts and purity of GST-Norbin and GST proteins produced, aliquots of beads were analyzed by SDS-PAGE and Coomassie staining alongside BSA standards. Typical amounts obtained were 3.3 pmol of purified GST-Norbin/μl of beads and 400 pmol of purified GST/μl of beads, and the purities of both proteins exceeded 97% in all preparations. For P-Rex1/Norbin interaction assays, washed glutathione-Sepharose 4B was added to GST beads to adjust their protein concentration to that of the GST-Norbin beads. For Rac-GEF assays, GST-Norbin and GST were eluted from the beads. Beads were washed 3 times in 50 mm Tris-HCl (pH 8.0), 150 mm NaCl, and 1 mm EGTA to remove detergent, and protein was eluted by incubation with an equal volume of 50 mm Tris-HCl (pH 8.0), 150 mm NaCl, 1 mm EGTA, and 20 mm glutathione for 10 min at 37 °C. Beads were sedimented, the supernatant recovered, and the elution repeated once, except with 10 mm glutathione. Both elutions were pooled. GST-Norbin was concentrated using an Ultracel Centricon device with a 50 molecular mass cut-off. The concentration of purified eluted GST-Norbin and GST proteins was determined by SDS-PAGE and Coomassie staining alongside BSA standards.

##### Direct Interaction of EE-P-Rex1 and GST-Norbin

Ten picomoles of purified recombinant human GST-Norbin or GST, immobilized on 3 μl of glutathione-Sepharose 4B, as described above, or glutathione-Sepharose 4B beads alone, were blocked in PBS, 50% FBS, 1% Triton X-100, 5 mm EGTA, 1 mm EDTA, and 1× mini-protease inhibitors, for 45 min at 4 °C with end-to-end rotation and washed once in PBS, 1% Triton X-100, 5 mm EGTA, 1 mm EDTA, 1× mini-protease inhibitors prior to incubation with 20 pmol of purified recombinant full-length human EE-P-Rex1 protein in PBS, 30% FBS, 1% Triton X-100, 5 mm EGTA, 1 mm EDTA, and 1× mini-protease inhibitors (*i.e.* the molar ratio of GST-Norbin or GST to EE-P-Rex1 being 1:2) in a volume of 300 μl for 90 min at 4 °C with end-to-end rotation. Beads were washed 4 times in PBS, 1% Triton X-100, 5 mm EGTA, 1 mm EDTA, and 1× mini-protease inhibitors. Bound proteins were denatured by boiling in Laemmli sample buffer, resolved on SDS-PAGE gels, and analyzed by Western blotting with P-Rex1 6F12, EE, and GST antibodies. Experiments with mutant EE-P-Rex1 proteins were performed the same way, except that 4.5 pmol of GST-Norbin or GST were incubated with 4.5 pmol of EE-P-Rex1 protein, and Western blotting was done with EE antibody.

##### Rac-GEF Activity in Vitro

This assay was carried out as described previously (1, 48). Briefly, it measures the exchange of GDP bound to recombinant purified Sf9 cell-derived human EE-Rac1 for GTPγS (and [^35^S]GTPγS) by purified recombinant human EE-P-Rex1. It was carried out in the presence of liposomes and, where appropriate, 10 μm synthetic stearoyl-arachidonyl PIP_3_ (1) or purified recombinant 0.3 μm EE-Gβ1γ2 proteins (1) as activators of P-Rex1. GST-Norbin or GST were incorporated by a 10-min preincubation with EE-P-Rex1 at 4 °C, at a molar ratio of 4:1, prior to the start of the assay. Samples containing EDTA were added as a control to determine the maximal GTPγS loading of EE-Rac1.

##### Pak-CRIB Pulldown

GST-Pak-CRIB immobilized on glutathione-Sepharose 4B was prepared as described (49) and stored in cell lysis buffer at 4 °C for up to 1 week. Ninety-millimeter dishes of HEK-293 cells were transfected as described above to overexpress EE-P-Rex1 and/or myc-Norbin, or mock-transfected. After 48 h, the cells were serum-starved in DMEM for 6 h and then stimulated with 50 nm lysophosphatidic acid (LPA) in DMEM for 2 min at 37 °C, or mock-treated with DMEM. To isolate GTP-loaded endogenous Rac1, the medium was aspirated and cells were scraped into 1.2 ml of ice-cold cell lysis buffer. Cells were lysed by incubation on ice for 5 min, with occasional vortexing, and insoluble material was removed by centrifugation at 16,000 × *g* for 5 min at 4 °C. Seventy-five microliters of each supernatant were retained as a total lysate control for Western blotting, and the remainder was incubated with 10 μl of GST-PAK-CRIB beads for 15 min on ice, with end-over-end rotation. Beads were washed 3 times with ice-cold cell lysis buffer, and proteins were denatured in boiling Laemmli sample buffer, resolved by SDS-PAGE and Western blotted with Rac1, P-Rex1 6F12, and Norbin C1 antibodies.

##### Immunofluorescence Microscopy

PAE cells were transfected to overexpress full-length or mutant eGFP-P-Rex1 and/or myc-Norbin as described above, or were mock-transfected, and grown on sterile 22-mm glass coverslips. Twenty-four hours after transfection, or 40 h for assays with mutants, cells were washed 3 times in Ham's F-12, serum-starved in Ham's F-12, 0.5% fatty-acid free BSA for 6 h, and then stimulated by the addition of prewarmed FBS to 10% final concentration, or with 10 ng/ml of PDGF or 2 μg/ml of LPA, for 5 min at 37 °C. Cells were fixed in 4% paraformaldehyde, 100 mm PIPES (pH 7.2; KOH), 2 mm EGTA, and 2 mm MgCl_2_ for 15 min followed by 3 washes in PBS and permeabilization in PBS, 0.2% Triton X-100 for 10 min and 3 more washes in PBS. After blocking in PBS, 0.5% BSA for 30 min, cells were stained with myc antibody for 1 h at room temperature, samples were washed twice in PBS and once in PBS, 0.5% BSA before incubation with Alexa Fluor 568 goat anti-mouse antibody (Invitrogen) in the dark for 30 min. Where appropriate, cells were also stained with Alexa 594-labeled phalloidin (Molecular Probes) for 20 min and/or with Hoechst (Sigma) DNA stain for 5 min. Coverslips were washed twice in PBS and once in Milli-Q water and mounted using Aqua Polymount (PolySciences). Samples were blinded and imaged using the ×60 (oil-immersion) or ×40 (air) objective lenses of a Zeiss Axiophot 2 fluorescence microscope. At least 100 transfected cells per coverslip were analyzed for the subcellular localization of eGFP-P-Rex1 and/or myc-Norbin and for the presence of lamellipodia and membrane ruffles. For super-resolution structured illumination microscopy (SIM), PAE cells were treated the same except that they were plated onto 18-mm high-performance Zeiss glass coverslips. Images were acquired using a Nikon dual mode SIM/STORM super resolution microscope and processed using Nikon Elements software.

##### Cell Fractionation

Ninety-millimeter dishes of HEK-293 cells were transfected to overexpress EE-P-Rex1 and/or myc-Norbin, or mock-transfected with empty vector, as described above, serum-starved for 6 h in DMEM, recovered by trypsinization, sedimented at 320 × *g* for 5 min, and resuspended in 1.0 ml of ice-cold HEK-293 cell fractionation buffer (40 mm HEPES, pH 7.4 at 4 ºC, 150 mm NaCl, 1 mm EDTA, 5 mm EGTA, Complete protease inhibitor mixture (Roche)). Cells were lysed by probe sonication using a Misonix 3000 sonicator with four 15-s on/off pulses of the microprobe on ice. One-hundred microliters of the total lysate were retained as a control for Western blotting. The remainder were subjected to centrifugation at 400 × *g* for 10 min at 4 °C to remove cell debris and nuclei. One-hundred microliters of the postnuclear supernatant were retained as a control for Western blotting and the remainder separated by ultracentrifugation at 100,000 × *g* for 60 min at 4 °C into cytosol and membrane fractions. Membrane pellets were washed twice gently in HEK-293 cell fractionation buffer. Proteins from all stages of the cell fractionation were denatured by boiling in Laemmli sample buffer (1× final concentration) as soon as each became available during the process. Samples were subjected to Western blotting with P-Rex1 6F12 and Norbin C1 antibody, followed by ImageJ densitometric analysis to quantify the amount of P-Rex1 and Norbin protein in each fraction.

## Results

### 

#### 

##### Identification of Norbin as a P-Rex1-binding Protein

**FIGURE 1.**
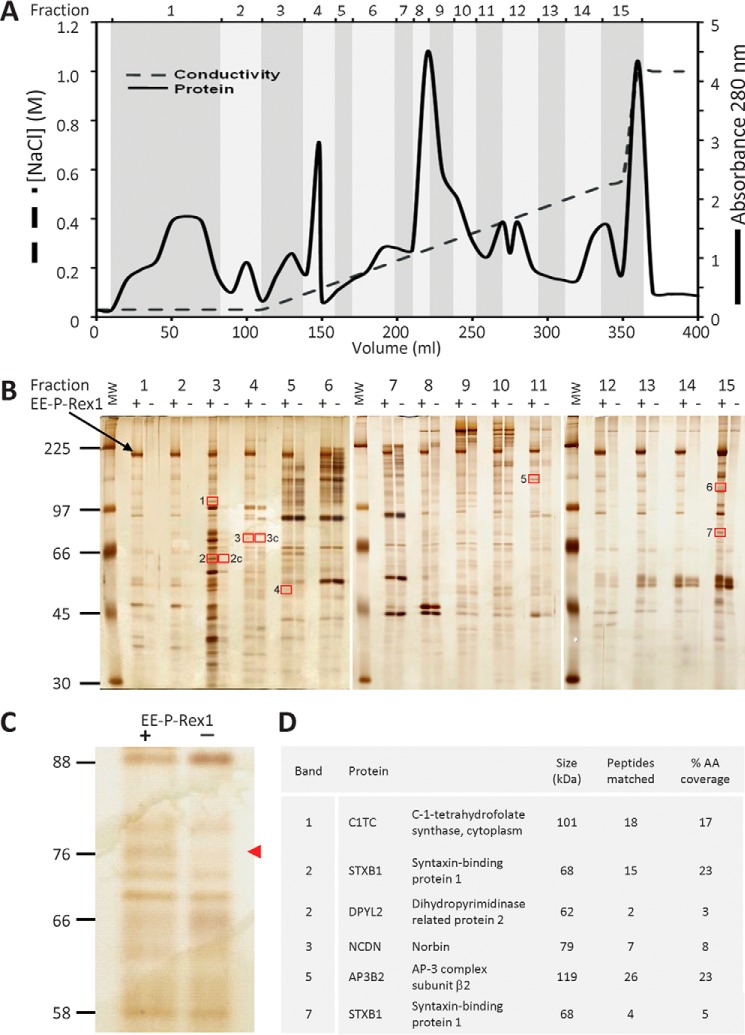
**Identification of Norbin as a P-Rex1-binding protein.** *A,* fractionation of P-Rex1^−/−^ mouse brain cytosol. Desalted P-Rex1^−/−^ mouse brain cytosol was fractionated by Source 15Q anion exchange chromatography with a linear 0.05–0.5 m NaCl gradient and a step to 1 m NaCl. 15 fractions ranging from the flow-through to the 1 m NaCl step and from 10 to 50 ml in volume were collected as indicated, and adjusted to 150 mm NaCl. *B,* isolation of putative P-Rex1-binding proteins from P-Rex1^−/−^ mouse brain cytosol fractions. The salt-adjusted fractions from *A* were pre-cleared and half of each incubated with 10 μg of purified recombinant human EE-P-Rex1 immobilized on EE antibody-coupled protein G-Sepharose (48), the other mock-treated with EE antibody beads alone. Bound proteins were subjected to SDS-PAGE and silver staining. Protein bands seen in the presence but not absence of EE-P-Rex1, framed by *red boxes*, and those labeled “*c*” as controls, were subjected to LC/MS. The *black arrow* highlights full-length EE-P-Rex1. The *vertical white lines* show boundaries between individual gels. *C*, enlargement of fraction 4 from the silver gel in *B*. The *red arrow* highlights the band identified as Norbin by LC/MS. *D,* list of putative P-Rex1 binding proteins identified. The numbering of bands corresponds to the *red-framed boxes* shown in *B*. Bands 4 and 6 contained only keratin and trypsin, so were not considered further.

Remarkably few binding partners of the Rac-GEF P-Rex1 have been discovered to date, which prompted us to search for more. As P-Rex1 is abundant in neuronal tissues, we decided to search for binding proteins in mouse brain, using purified recombinant EE-tagged P-Rex1 protein (1) as bait. We reasoned that P-Rex1-binding proteins might be more likely to coimmunoprecipitate with EE-P-Rex1 in the absence of competition from endogenous P-Rex1, and therefore used brain tissue from P-Rex1^−/−^ mice as the source. Furthermore, as P-Rex1 is largely cytosolic, we decided to aim for cytosolic binding partners. We prepared soluble proteins (detergent-free lysate) from P-Rex1^−/−^ mouse brain and fractionated them by anion exchange chromatography into 15 fractions using a salt gradient (Fig. 1*A*). Each fraction was adjusted to physiological salt concentration and subjected to coimmunoprecipitation with purified recombinant EE-P-Rex1 protein that had been immobilized on EE-antibody coupled protein G-Sepharose, using mock treatment with EE-antibody beads as a control. Bound proteins were resolved by SDS-PAGE and visualized by silver staining (Fig. 1, *B* and *C*). Seven protein bands that were visible in the presence but not absence of EE-P-Rex1 (and two control bands) were excised for further analysis, digested with a range of proteases, and analyzed by mass spectrometry. Five potential P-Rex1-binding proteins were identified in this manner: the vesicle trafficking proteins STXB1 and AP3B2, the metabolic proteins DPYL2 and C1TC, and the GPCR adaptor Norbin (Fig. 1*D*). Three of these were found in fraction 3: C1TC in band 1, STXB1 and DPYL2 in band 2, all migrating as expected for their *M*_r_, and STXB1 was also found in fraction 15, band 7. Other bands in fraction 3 were not analyzed, because careful evaluation of the surrounding lanes suggested that they likely contained proteins that bound nonspecifically to the beads. Norbin was recovered in fraction 4, band 3, and AP3B2 in fraction 11, band 5. Bands 4 and 6 contained only keratin and trypsin, so were not considered further.

Among the five proteins identified, we selected Norbin for further characterization, for several reasons: Norbin coimmunoprecipitated with EE-P-Rex1 at its expected full-length molecular mass of 79 kDa (Fig. 1*C*); Norbin is abundant in neurons and largely cytosolic, meaning its tissue and subcellular distribution overlap with those of P-Rex1; like P-Rex1, Norbin affects neuronal morphology and plasticity; and finally, like P-Rex1, Norbin affects GPCR signaling, albeit through different mechanisms.

##### Norbin Tissue Distribution

**FIGURE 2.**
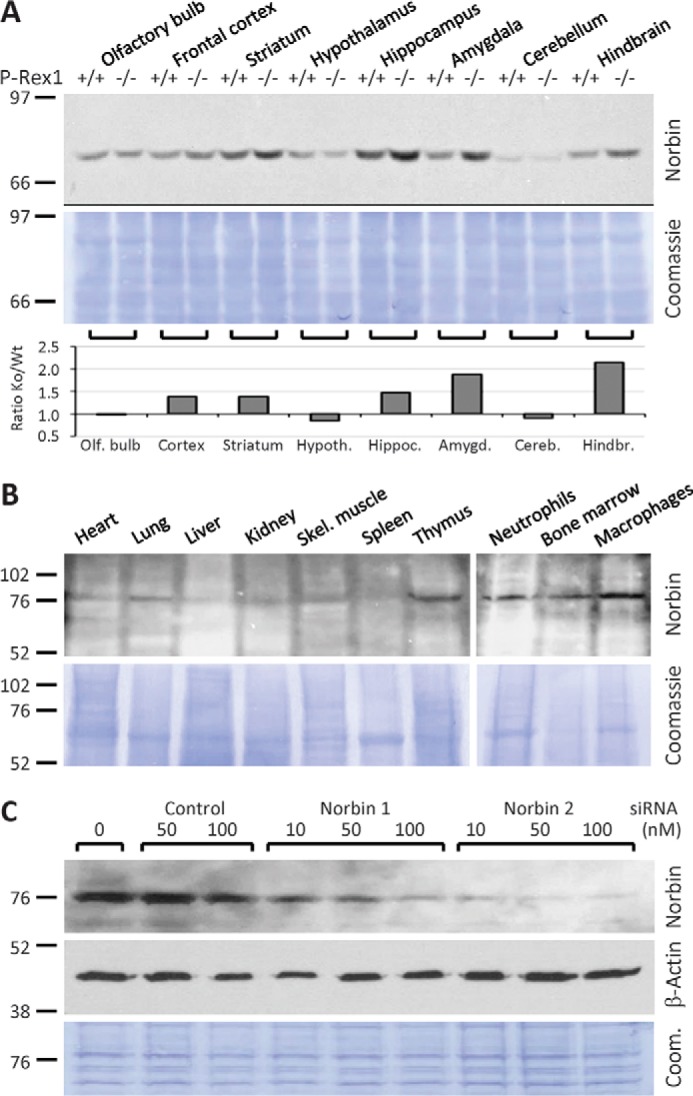
**Norbin tissue distribution.** *A,* Norbin distribution in the brain and up-regulation in some regions of P-Rex1^−/−^ mouse brain. Total lysates of brain sections from adult P-Rex1^+/+^ (Wt) and P-Rex1^−/−^ (Ko) mice (6, 10) were Western blotted and Norbin levels quantified by ImageJ and expressed as the ratio of P-Rex1^−/−^ to corresponding P-Rex1^+/+^ samples. *B*, Norbin distribution in non-neuronal mouse tissues and cell types. Total lysates of organs, bone marrow cells, macrophages, and neutrophils isolated from adult wild-type mice (100 μg of tissue per lane) were Western blotted for Norbin expression. The *vertical white lines* show boundaries between individual gels. *C*, test of Norbin antibody. To confirm the specificity of the Norbin antibody, HEK-293 cells were treated with the indicated concentrations of siRNA1 and siRNA2 to down-regulate endogenous Norbin or a pool of 4 non-targeting siRNAs as a control. Norbin C1 antibody was used in *A–C* to detect endogenous Norbin, and β-actin antibody or Coomassie staining of the membranes to control for total protein loading.

Prior to characterizing the interaction between P-Rex1 and Norbin, we evaluated the tissue distribution of Norbin further, reasoning that if their interaction were important for function, Norbin might be expressed in more tissues than the previously reported nervous system, cartilage, and bones (33, 35, 37, 38). We obtained a panel of polyclonal Norbin antisera (N4 and N7 raised against the N-terminal residues Met^1^-Ile^17^ of Norbin; C1 and C3 against the C-terminal residues Thr^713^-Pro^729^) that had previously been shown to recognize Norbin in Western blotting, immunofluorescence, and immunohistochemistry applications (kind gifts from Prof. Kei Maruyama, Tokyo Institute of Psychiatry, Japan) (33, 38, 44). We used Norbin C1 antibody to verify the expected widespread distribution of Norbin in the brain and assess if P-Rex1 deficiency affects Norbin levels, by Western blotting lysates from various regions of the brain from adult P-Rex1^+/+^ and P-Rex1^−/−^ mice (10). Norbin was detected in all brain tissues tested, as expected. Interestingly, Norbin levels were increased 2-fold in the amygdala and hindbrain of P-Rex1^−/−^ mice, and up to 1.5-fold in the hippocampus, frontal cortex, and striatum (Fig. 2*A*). These data confirmed that Norbin is expressed throughout the brain, as expected, similarly to P-Rex1 (10), and they suggest furthermore, that Norbin may be up-regulated to compensate for the P-Rex1 deficiency.

To test how widely Norbin is expressed in other tissues, we used Norbin C1 antibody for Western blotting lysates from a number of organs and primary cell types of adult wild-type mice. A band of 79 kDa was detected in most tissues, including heart, lung, kidney, and skeletal muscle, although not in spleen, and barely in liver. Signals were especially clear in thymus, bone marrow cells, bone marrow-derived mature neutrophils, and peritoneal macrophages (Fig. 2*B*). Although these signals were lower than in the brain, they suggested that the tissue distribution of Norbin, and by inference its functional roles, may be more widespread than previously appreciated.

To verify that the 79-kDa band detected by the Norbin C1 antibody was indeed Norbin, we down-regulated endogenous Norbin from HEK-293 cells using two independent Norbin siRNAs (Dharmacon). 50 nm siRNA2 knocked the 79-kDa band down by 95% and siRNA1 by 80%, whereas a pool of 4 non-targeting control siRNAs had no effect at this concentration (Fig. 2*C*). Therefore, the 79-kDa band recognized by Norbin C1 antibody in HEK-293 cells, and in the multiple tissue and hematopoietic cell blots, was confirmed as endogenous Norbin. These data show that the tissue distribution of Norbin is more widespread than previously appreciated. They suggest furthermore, that P-Rex1 and Norbin may be functionally linked not just in neurons but in also in other cell types, including myeloid cells such as neutrophils, where P-Rex1 is known to play important functional roles (3–6, 9).

##### P-Rex1 and Norbin Interact Directly and in Cells

**FIGURE 3.**
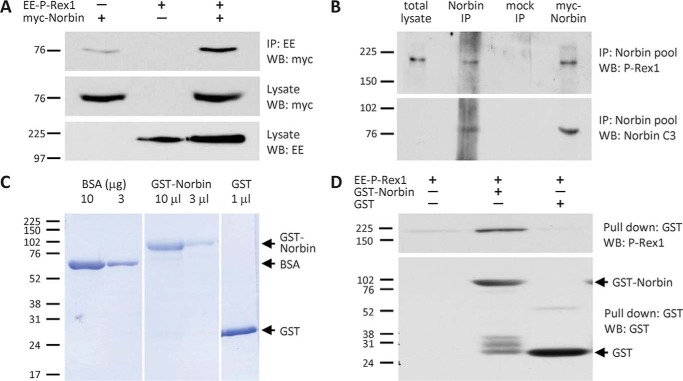
**Norbin binds P-Rex1 directly and interacts with P-Rex1 endogenously in cells.** *A,* myc-Norbin interacts with EE-P-Rex1. COS-7 cells expressing EE-P-Rex1 and/or myc-Norbin were subjected to immunoprecipitation with immobilized EE antibody under stringent conditions (1% Triton X-100) and analyzed by Western blotting with myc and EE Abs as indicated. Blots shown are from 1 experiment representative of 3. *B*, endogenous P-Rex1 and Norbin interact. HEK-293 cell lysates were incubated with Norbin antibody pool (C1, N4, N7) or mock-treated with serum-IgG, washed stringently and Western blotted. Overexpression of myc-Norbin was used as a control. Blots shown are from 1 experiment representative of 3. *C*, purification of GST-Norbin. Coomassie gel showing purified recombinant GST-Norbin and GST (bead volumes are indicated) and BSA standards. The *vertical white lines* denote cropped lanes. *D,* direct interaction between EE-P-Rex1 and GST-Norbin. 10 pmol of immobilized purified recombinant GST-Norbin or GST, or beads alone, were incubated with 20 pmol of purified recombinant EE-P-Rex1, beads were washed stringently, and proteins were analyzed by Western blotting. Blots shown are from 1 experiment representative of 3.

To confirm our initial identification of Norbin as a P-Rex1-binding protein, we overexpressed EE-P-Rex1 and/or myc-Norbin in COS-7 cells and used EE-antibody beads to immunoprecipitate EE-P-Rex1 from cell lysates. Western blotting showed that myc-Norbin coimmunoprecipitates with EE-P-Rex1, whereas it bound to EE-antibody beads alone only weakly. These results confirmed that exogenously expressed P-Rex1 and Norbin interact within cells (Fig. 3*A*). To assess whether endogenous P-Rex1 can also interact with endogenous Norbin, we used HEK-293 cells, which express both proteins endogenously. We used a pool of Norbin antibodies (C1, N4, N7) to immunoprecipitate endogenous Norbin from HEK-293 cell lysates under stringent detergent conditions and analyzed P-Rex1 binding by Western blotting with P-Rex1 6F12 (6) and Norbin C3 (33) antibodies. These experiments revealed that endogenous P-Rex1 interacts with endogenous Norbin in HEK-293 cells (Fig. 3*B*).

To assess whether P-Rex1 and Norbin can interact directly, we carried out *in vitro* binding assays with Sf9 cell-derived purified recombinant EE-P-Rex1 protein, prepared as previously described (18, 48), and bacterially produced purified recombinant GST-Norbin and GST proteins (Fig. 3*C*). For these assays, GST-Norbin was immobilized on glutathione-Sepharose, and immobilized GST or beads alone were used as controls. Twenty pmol of EE-P-Rex1 were incubated with 10 pmol of immobilized GST-Norbin or GST, beads were washed stringently, and protein binding analyzed by Western blotting with P-Rex1 6F12 and GST antibodies. EE-P-Rex1 binding to GST-Norbin was readily detected whereas, nonspecific binding of P-Rex1 to immobilized GST or to beads alone was seen at low levels (Fig. 3*D*). Together, these data show that Norbin is a direct P-Rex1-binding protein that also interacts with P-Rex1 endogenously in cells.

##### P-Rex1 Binds Norbin via Its PH Domain

**FIGURE 4.**
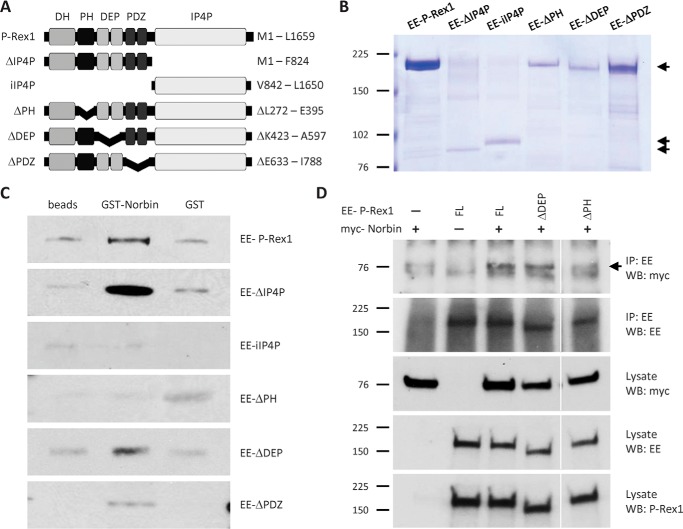
**The PH domain of P-Rex1 is required for Norbin binding.** *A,* schematic of P-Rex1 mutants. *B,* purified mutant P-Rex1 proteins. Coomassie-stained SDS-PAGE showing 100 pmol of recombinant full-length or mutant EE-P-Rex1 proteins (50 pmol for EE-IP4P), purified from Sf9 cells. *C,* the PH domain of P-Rex1 is required for Norbin binding *in vitro*. 4.5 pmol of purified full-length or mutant EE-P-Rex1 proteins were incubated with 4.5 pmol of purified GST-Norbin or GST, or with beads alone, and Western blotted with EE antibody. Blots shown are from 1 experiment representative of 3 for the full-length protein and for ΔPH, ΔDEP, and ΔPDZ mutants and 5 for ΔIP4P and iIP4P. *D,* the PH domain of P-Rex1 is required for the interaction with Norbin *in vivo*. COS-7 cells expressing combinations of myc-Norbin full-length (*FL*) EE-P-Rex1, EE-ΔDEP, or EE-ΔPH were immunoprecipitated with immobilized EE antibody under stringent conditions (RIPA buffer) and analyzed by Western blotting with myc-HRP, EE, and P-Rex1 6F12 ABs. The *white vertical lines* show a cropped lane. Blots shown are from 1 experiment representative of 4.

To characterize which P-Rex1 domains confer the interaction with Norbin, we measured direct binding of full-length GST-Norbin to a panel of EE-tagged truncation or deletion mutants of P-Rex1 that were produced by baculoviral expression in Sf9 insect cells and purified as previously described (18, 48) (Fig. 4, *A* and *B*). The panel consisted of full-length P-Rex1, the isolated IP4P domain (iIP4P), and mutants lacking the PH (ΔPH), DEP (ΔDEP), PDZ (ΔPDZ), and IP4P (ΔIP4P) domains. The functional integrity of these mutant EE-P-Rex1 proteins was validated previously. They all possess Rac-GEF activity (except the iIP4P mutant, which lacks the catalytic domain), and their Rac-GEF activity is stimulated by Gβγ subunits and PIP_3_ (the latter except the ΔPH mutant, as PIP_3_ binds to the PH domain) (18, 48). Binding assays with immobilized GST-Norbin (using immobilized GST and beads alone as controls) were carried out as in Fig. 3*D*, except that 4.5 pmol of EE-P-Rex1 proteins were incubated with 4.5 pmol of GST-Norbin or GST, and Western blotting was done with anti-EE antibody, to enable equal detection of all mutants. Preferential binding to immobilized GST-Norbin over GST or beads alone was seen with full-length EE-P-Rex1 and with the ΔDEP, ΔPDZ, and ΔIP4P mutants, but not with the iIP4P or ΔPH mutants which, if at all, bound nonspecifically to immobilized GST or beads alone (Fig. 4*C*). These data show that the N-terminal half of P-Rex1 is required and sufficient for direct Norbin binding and that, within the N-terminal half, the PH domain of P-Rex1 is required. It should also be noted that binding of GST-Norbin to the ΔPDZ mutant was weak and not seen in all experiments, suggesting that the PDZ domains of P-Rex1 might also contribute to Norbin binding.

To study the requirement of P-Rex1 domains for the interaction with Norbin *in vivo*, we performed coimmunoprecipitation assays in COS-7 cells as in Fig. 3*A*, but by co-expressing myc-Norbin either with full-length or mutant forms of EE-P-Rex1. We tested one mutant that could bind Norbin directly *in vitro*, ΔDEP, and one mutant that could not, ΔPH. The EE-ΔDEP mutant coimmunoprecipitated with myc-Norbin as did full-length EE-P-Rex1, whereas the EE-ΔPH mutant did not (Fig. 4*D*). These results show that the PH domain of P-Rex1 is required for Norbin binding *in vivo*, suggesting that similar P-Rex1 domains confer the interaction with Norbin *in vitro* and *in vivo*.

##### Norbin Stimulates the Rac-GEF Activity of P-Rex1 in Vitro and in Cells

**FIGURE 5.**
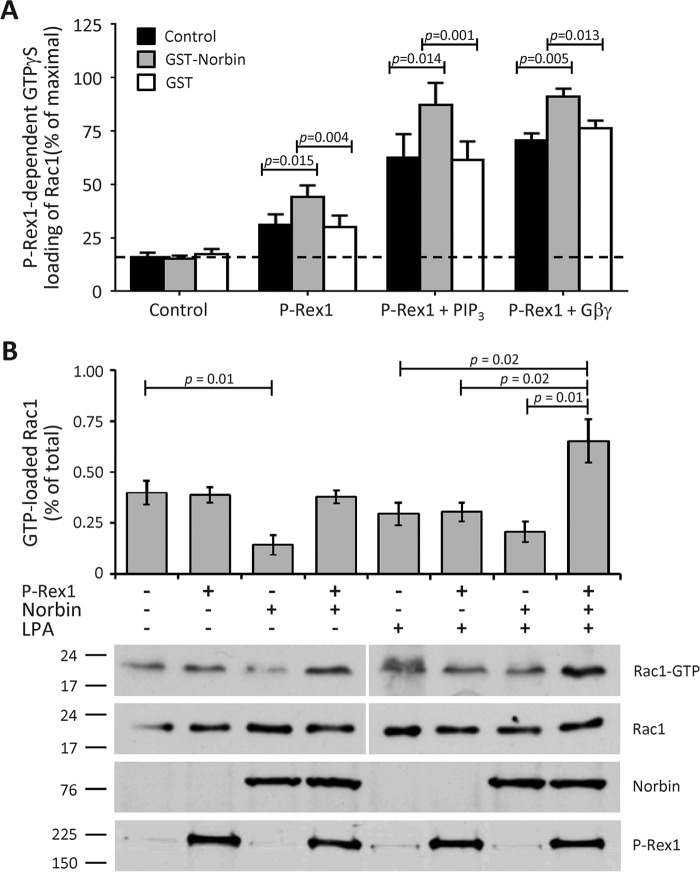
**Norbin stimulates the Rac-GEF activity P-Rex1.** *A*, GST-Norbin directly stimulates the basal, PIP_3_- and Gβγ-dependent Rac-GEF activity of EE-P-Rex1. The ability of 50 nm purified full-length EE-P-Rex1 to activate (GTPγS-load) 100 nm EE-Rac1 within 10 min was tested ±200 nm purified GST-Norbin or GST protein, and with liposomes ±10 μm stearoyl-arachidonyl PIP_3_ or 0.3 μm Gβ_1_γ_2_, as indicated. Rac-GEF activity is expressed as % of EE-Rac1-GTPγS in EDTA controls. Data are mean ± S.E. of 10 independent experiments testing basal activity, 5 with PIP_3_ and 6 with Gβ_1_γ_2_; statistics are paired *t* test with Bonferroni/Holm multiple comparisons analysis. *B*, coexpression of P-Rex1 and Norbin activates endogenous Rac1 in LPA-stimulated HEK-293 cells. HEK-293 cells expressing EE-P-Rex1 and/or myc-Norbin, or mock-transfected cells, were serum-starved, stimulated with 50 nm LPA for 2 min, or mock-stimulated, and subjected to Pak-CRIB pulldown and Western blotting (1% of cell lysate loaded for total Rac1). Blots shown are from 1 experiment representative of 4. The *vertical white lines* show boundaries between gels run and blotted together, with the corresponding Rac1-GTP and total lysate Rac1 samples on one gel. Quantification of Rac1 activity in mock-stimulated (*left-hand panel*) and LPA-stimulated cells (*right-hand panel*) is expressed as mean ± S.E. of 4 experiments; statistics are unpaired *t* test.

To test whether the direct interaction between P-Rex1 and Norbin affects the Rac1-GEF activity of P-Rex1, purified GST-Norbin (as in Fig. 3, *C* and *D*, except not immobilized) was incorporated into a liposome-based *in vitro* Rac-GEF activity assay that measures the activation (GTPγS loading) of Rac by P-Rex1, in the presence or absence of P-Rex1 activators PIP_3_ and Gβγ (1, 48). Here, we used Sf9 cell-derived purified recombinant prenylated GDP-loaded EE-Rac1 and EE-Gβ_1_γ_2_ proteins, full-length EE-P-Rex1 (as in Fig. 3, *C* and *D*), and synthetic stearoyl-arachidonyl PIP_3_. In the absence of EE-P-Rex1, 16% of Rac1 was active (GTPγS loaded), and GST-Norbin or GST had no effect on this basal activity, whereas EE-P-Rex1 increased Rac1 activity to 31%. The combined addition of EE-P-Rex1 and GST-Norbin at a molar ratio of 1:4 increased Rac1 activity to 44%, whereas GST again had no effect. These data show that GST-Norbin directly stimulates the basal Rac-GEF activity of P-Rex1. PIP_3_ and Gβγ proteins also stimulated the activity of P-Rex1, as expected, and GST-Norbin increased this further from 63 to 87% and from 71 to 91%, respectively, whereas GST again had no effect (Fig. 5*A*). Therefore, Norbin directly stimulates the basal, PIP_3_- and Gβγ-dependent Rac1-GEF activities of P-Rex1, modestly but significantly. The degree of P-Rex1 activation by Norbin was similar under all conditions, roughly additive to the effects of PIP_3_ and Gβγ. This suggests that Norbin binding might increase the availability of P-Rex1 in the liposomes that contain the prenylated Rac1 and Gβγ proteins and the PIP_3_ phospholipid, thereby bringing P-Rex1 into closer contact with its substrate and its other activators.

To assess whether Norbin can also stimulate the Rac-GEF activity of P-Rex1 *in vivo*, we performed Pak-CRIB pulldown assays (49). HEK-293 cells overexpressing EE-P-Rex1 and/or myc-Norbin, or mock-transfected cells as controls, were serum-starved and stimulated with the GPCR ligand LPA, which is known to stimulate GPCR-mediated P-Rex1 activation in HEK-293 cells (23), or they were mock-stimulated. Endogenous active (GTP-bound) Rac1 was isolated from total cell lysates using GST-Pak-CRIB immobilized on glutathione-Sepharose, and GTP-Rac1 and total Rac1 levels were analyzed by Western blotting. In basal (serum-starved) cells, 0.40% of the total cellular Rac1 was active, and the expression of myc-Norbin significantly reduced this level of active Rac1 to 0.14%. As we aimed for a modest level of overexpression, EE-P-Rex1 unsurprisingly did not affect Rac1 activity in these basal cells, nor did coexpression with myc-Norbin (the slight increase in the blots shown was not observed consistently in all experiments). However, upon cell stimulation with 50 nm LPA for 2 min, coexpression of EE-P-Rex1 and myc-Norbin caused an increase in active Rac1 to 0.65% of the total cellular protein (Fig. 5*B*). These results suggest that Norbin stimulates the Rac1-GEF activity of P-Rex1 upon activation of HEK-293 cells through GPCRs. Therefore, the interaction of P-Rex1 and Norbin has functional importance in GPCR signaling. Furthermore, compared with the modest Norbin-dependent stimulation of P-Rex1 Rac-GEF activity in the liposome *in vitro* assay, the effect in HEK-293 cells was quite robust, suggesting that the *in vitro* assay does not completely recapitulate the complexity of the P-Rex1/Norbin interaction *in vivo*.

##### Norbin and P-Rex1 Promote Plasma Membrane Localization in Each Other and Stimulate Rac1 Activity-dependent Cell Morphologies

In addition to stimulating the Rac-GEF activity of P-Rex1, Gβγ and PIP_3_ also regulate the protein by synergistically driving its translocation from the cytosol to the plasma membrane (26, 27). To assess whether Norbin also affects the subcellular localization of P-Rex1, we carried out immunofluorescence microscopy in PAE cells. In addition, we assessed cell morphology. PAE cells have a kite-shaped morphology when basal, but adopt characteristic morphologies, namely lamellipodia formation, membrane ruffling, and cell spreading, in response to P-Rex1-dependent activation of Rac1, through the induction of actomyosin cytoskeletal dynamics (1, 50). PAE cell morphology thereby provides an indirect but sensitive readout of endogenous Rac1 activity that can be assessed on a cell-by-cell basis through imaging (2).

**FIGURE 6.**
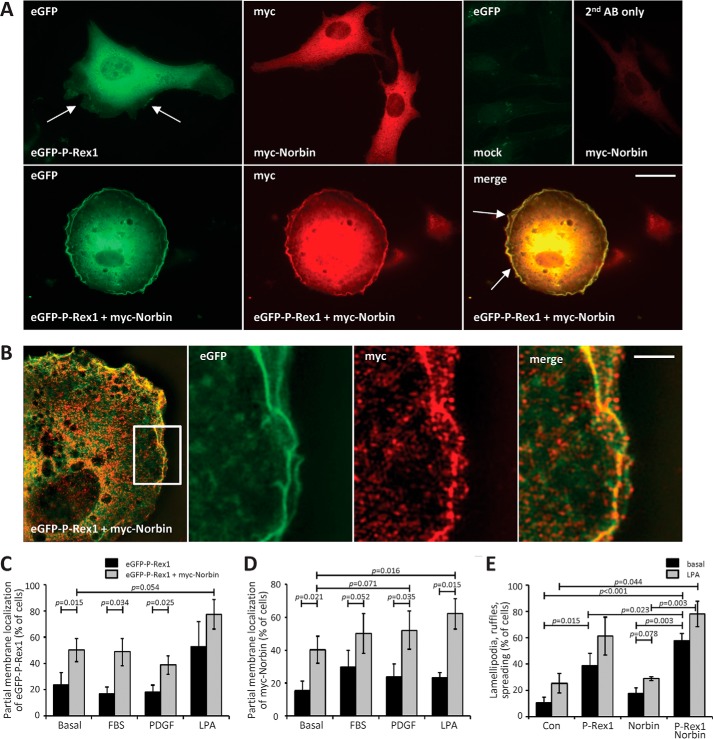
**P-Rex1 and Norbin promote the plasma membrane localization of each other and elicit cell spreading, lamellipodia formation and membrane ruffling.** *A,* immunofluorescence micrographs. PAE cells expressing eGFP-P-Rex1 and/or myc-Norbin for 24 h were serum-starved for 6 h, fixed, permeabilized, and stained with myc antibody and Alexa Fluor 568 goat anti-mouse secondary antibody, or with secondary antibody alone, and subjected to widefield immunofluorescence microscopy. *Arrows* indicate lamellipodia and membrane ruffles. The *scale bar* represents 10 μm. Representative images from 1 of 4 independent experiments are shown. *B*, super-resolution structured illumination micrographs. Representative super-resolution SIM image of part of a PAE cell expressing eGFP-P-Rex1 and myc-Norbin, serum-starved and stained as in *A* (*left-hand panel*), with a zoom (*white box*) into membrane ruffles at the cell edge. One Z-plane of 0.12 μm depth is shown. The *scale bar* represents 2 μm. *C–E*, quantification of immunofluorescence microscopy. PAE cells expressing eGFP-P-Rex1 (*black bars*) or eGFP-P-Rex1 and myc-Norbin (*gray bars*) as in *A*, or mock-transfected cells, were serum-starved for 6 h and stimulated with 10% FBS, 10 ng/ml of PDGF, or 2 μg/ml of LPA for 5 min, or mock-treated, and stained as in *A*. Slides were blinded and analyzed by imaging for the percentage of cells showing partial membrane localization of eGFP-P-Rex1 (*C*) or myc-Norbin (*D*). *E*, co-expression of P-Rex1 and Norbin induces cell morphologies characteristic of active Rac1. Basal (serum-starved; *black bars*) or LPA-stimulated (*gray bars*) PAE cells expressing eGFP-P-Rex1 and/or myc-Norbin as in (*C* and *D*) were evaluated for the occurrence of lamellipodia, membrane ruffles, and cell spreading. In *C–E*, ≥100 transfected cells were scored per coverslip, with duplicate coverslips per condition and experiment. Data are mean ± S.E. from 7 independent experiments with basal cells, 3 with FBS and 4 with PDGF or LPA stimulation, respectively; statistics are paired *t* test with Bonferroni/Holm multiple comparisons analysis.

PAE cells expressing eGFP-P-Rex1 and/or myc-Norbin were serum-starved, fixed, stained, and the subcellular localizations of eGFP-P-Rex1 and myc-Norbin as well as cell shape were evaluated by widefield immunofluorescence imaging. The localization of eGFP-P-Rex1 appeared largely cytosolic when the Rac-GEF was expressed alone, although some cells showed a partial membrane localization of the GEF, with lamellipodia formation and membrane ruffling, as expected. myc-Norbin appeared also largely cytosolic when expressed alone, and these cells had a basal morphology. In contrast, coexpression of eGFP-P-Rex1 and myc-Norbin seemed to induce a translocation of both proteins to the plasma membrane, and stimulate lamellipodia, membrane ruffling, and cell spreading (Fig. 6*A*). To investigate if eGFP-P-Rex1 and myc-Norbin co-localize, we imaged these PAE cells using super-resolution SIM. This imaging confirmed that eGFP-P-Rex1 and myc-Norbin are largely localized in the cytoplasm, with eGFP-P-Rex1 showing a more homogenous distribution than myc-Norbin, which was slightly more punctate. Some colocalization of eGFP-P-Rex1 and myc-Norbin was seen in the cytoplasm. However, much more substantial colocalization of eGFP-P-Rex1 and myc-Norbin was observed in membrane ruffles at the cell edge (Fig. 6*B* and supplemental Movie S1).

We next assessed the effects of cell stimulation on the localizations of eGFP-P-Rex1 and myc-Norbin and on cell shape. PAE cells expressing eGFP-P-Rex1 and/or myc-Norbin were serum-starved and stimulated with 10% FBS, 10 ng/ml of PDGF or 2 μg/ml of LPA for 5 min, or were mock-stimulated, before being fixed and stained. The localization of eGFP-P-Rex1 and myc-Norbin was evaluated by widefield immunofluorescence imaging. eGFP-P-Rex1 was largely localized in the cytosol when expressed alone, but 24% of basal (serum-starved) PAE cells showed a partial membrane localization. Cell stimulation did not affect this localization, although there was a tendency for increased membrane localization upon LPA treatment that was not statistically significant. In contrast, coexpression of eGFP-P-Rex1 and myc-Norbin significantly increased the plasma membrane localization of P-Rex1 to 50% of cells under basal conditions, with similar increases upon FBS or PDGF stimulation and an increase to 78% upon LPA stimulation (Fig. 6*C*). Therefore, myc-Norbin promotes the plasma membrane localization of eGFP-P-Rex1 in PAE cells.

Like P-Rex1, myc-Norbin was also largely localized in the cytoplasm when expressed alone, but showed partial membrane localization in 16% of basal cells. Cell stimulation with FBS, PDGF, or LPA seemed to increase the membrane localization of myc-Norbin up to 2-fold, but as with eGFP-P-Rex1 this was insufficient to be statistically significant. In contrast, coexpression with eGFP-P-Rex1 significantly induced the plasma membrane localization of myc-Norbin to 40% of basal cells and to 50–64% of the various stimulated cells (Fig. 6*D*). Therefore, as well as Norbin promoting a robust plasma membrane translocation of P-Rex1, P-Rex1 inversely increases the plasma membrane localization of Norbin. In addition, in cells that coexpressed eGFP-P-Rex1 and myc-Norbin, LPA stimulation further enhanced the membrane localization of myc-Norbin and showed a tendency to also increase that of eGFP-P-Rex1 (Fig. 6, *C* and *D*).

As well as affecting the subcellular localizations of each other, the interaction of P-Rex1 and Norbin also controlled the morphology of PAE cells. Most mock-transfected serum-starved cells had the typical kite shape of basal PAE cells, whereas expression of eGFP-P-Rex1 alone induced lamellipodia formation, membrane ruffling, and cell spreading in 39% of the cells, as expected (1). Expression of myc-Norbin alone caused limited lamellipodia formation and membrane ruffling that did not reach statistical significance, and no cell spreading, which was unsurprising, as the latter requires high levels of Rac1 activity (50). Norbin-dependent lamellipodia formation and membrane ruffling would be consistent with its role in neurite outgrowth (33), which requires similar Rac1-dependent cytoskeletal dynamics (51), and it seems plausible that any limited effect seen here could be mediated through endogenous P-Rex1. In contrast, coexpression of eGFP-P-Rex1 and myc-Norbin increased the proportion of cells with lamellipodia, ruffles, and/or spreading significantly to 58% (Fig. 6*E*). These effects on cell morphology likely reflect the Norbin-dependent increase in P-Rex1 plasma membrane localization and Rac1-GEF activity. LPA stimulation showed a tendency to stimulate Rac1-dependent cell morphologies independently of the expression of eGFP-P-Rex1 or myc-Norbin, but this was a minor effect compared with that observed upon coexpression of eGFP-P-Rex1 and myc-Norbin (Fig. 6*E*).

**FIGURE 7.**
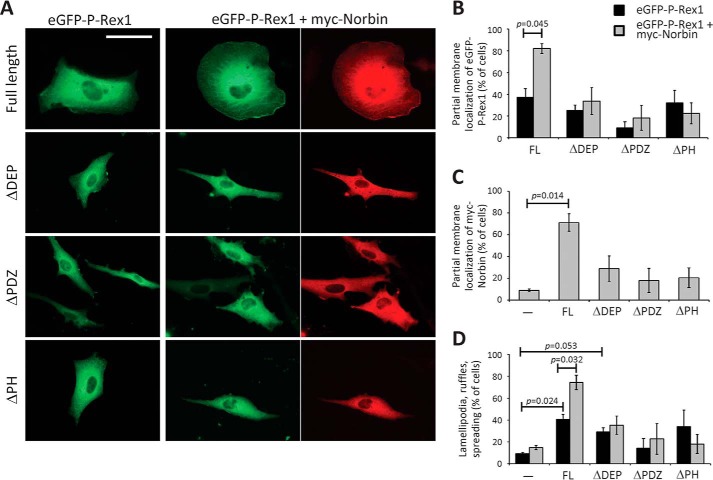
**P-Rex1 domains required for Norbin-dependent plasma membrane localization and effects on cell morphology.** *A*, immunofluorescence micrographs. PAE cells expressing full-length or mutant eGFP-P-Rex1, as indicated, with or without myc-Norbin for 40 h were serum-starved for 6 h, fixed, permeabilized, and stained with myc antibody and Alexa Fluor 568 goat anti-mouse secondary antibody, or with secondary antibody alone, and subjected to widefield immunofluorescence microscopy. The *scale bar* represents 20 μm. Representative images from 1 of 3 independent experiments are shown. *B–D*, quantification of immunofluorescence microscopy. PAE cells as in *A* expressing full-length (*FL*) or the indicated mutant forms of eGFP-P-Rex1 (*black bars*) or expressing these together with myc-Norbin (*gray bars*), or mock-transfected cells, were treated as described in *A*. Slides were blinded and analyzed for the percentage of cells showing partial membrane localization of eGFP-P-Rex1 (*B*), myc-Norbin (*C*), and cell morphologies (*D*) characteristic of active Rac1 (lamellipodia, membrane ruffles, and cell spreading). In *B–D*, ≥100 transfected cells were scored on duplicate coverslips per condition. Data are mean ± S.E. from 3 independent experiments; statistics are paired *t* test with Bonferroni/Holm multiple comparisons analysis.

To assess if the same P-Rex1 domains that confer Norbin binding are also required for the ability of Norbin to stimulate P-Rex1-dependent lamellipodia formation, membrane ruffling, and cell spreading, we performed a similar experiment as in Fig. 6, by comparing full-length eGFP-P-Rex1 with the eGFP-tagged mutants ΔDEP, ΔPDZ, and ΔPH in the presence or absence of myc-Norbin expression (Fig. 7*A*). To allow all forms of P-Rex1 to express to comparable levels, we cultured the cells for 40 h prior to serum starvation. Under these conditions, membrane localization of full-length eGFP-P-Rex1 was higher than in Fig. 6*C*, but it was stimulated by myc-Norbin to a similar degree (Fig. 7*B*), as was the eGFP-P-Rex1-dependent translocation of myc-Norbin to the membrane (Fig. 7*C*), and the ability of myc-Norbin to stimulate P-Rex1-dependent lamellipodia formation, membrane ruffling, and cell spreading (Fig. 7*D*). All P-Rex1 mutants were largely localized in the cytoplasm when expressed alone and did not affect cell morphology significantly, although eGFP-ΔDEP showed a tendency for stimulating lamellipodia, ruffling, and spreading (Fig. 7, *A–D*). myc-Norbin failed to induce the membrane localization of eGFP-ΔPH (or vice versa), which was expected, as experiments in Fig. 4 had shown that it could not bind EE-ΔPH *in vitro* or *in vivo*. Similar results were obtained with eGFP-ΔPDZ, which was also unsurprising considering that EE-ΔPDZ only weakly bound myc-Norbin *in vitro*. However, myc-Norbin also could not stimulate the membrane localization of eGFP-ΔDEP (or vice versa) or affect the morphology of these cells (Fig. 7, *A–D*), although it could bind EE-ΔDEP *in vitro* and *in vivo*. These results show that the entire N-terminal half of P-Rex1 is required for Norbin to stimulate its membrane localization and cell responses, a wider region than that required for Norbin binding. They suggest that additional cellular regulators exist that facilitate the membrane localization of both proteins and their effects on the cell.

**FIGURE 8.**
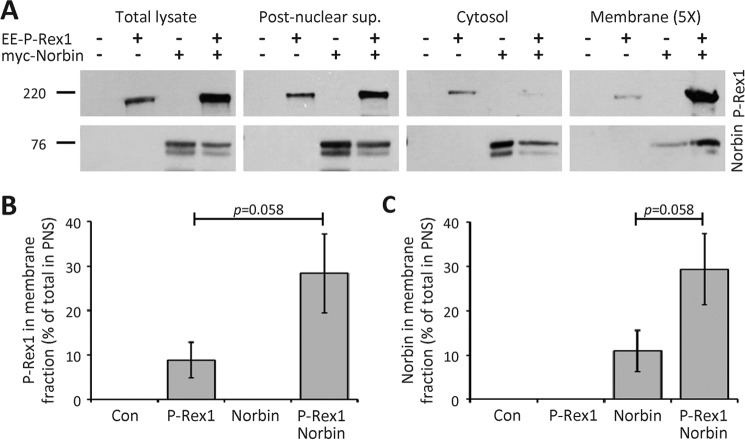
**P-Rex1 and Norbin promote the localization of each other in membrane fractions.** *A,* HEK-293 cells expressing EE-P-Rex1 and/or myc-Norbin, and mock-transfected cells, were serum-starved, lysed, the total lysates were cleared of debris and nuclei, and the postnuclear supernatant fractionated into membrane and cytosol fractions. Aliquots from all stages of the fractionation were analyzed by Western blotting with P-Rex1 6F12 and Norbin C1 antibodies. Note that 5 times as much membrane fraction was loaded than of the other fractions, to allow direct comparison. Blots shown are from 1 experiment representative of 3. Total lysates and postnuclear supernatant were run on one gel, cytosol and membrane fractions on another; the *vertical white lines* denote cropped lanes. *B* and *C,* Western blots as in *A* were quantified by ImageJ and the amount of P-Rex1 (*B*) and Norbin (*C*) in the membrane fraction expressed as percent of the postnuclear supernatant. Data are mean ± S.E. from 3 independent experiments; statistics are unpaired *t* test.

Together, our data in PAE cells showed that P-Rex1 and Norbin promote plasma membrane localization with each other and cooperate in stimulating Rac activity-dependent cell morphologies. To confirm in another cell type that P-Rex1 and Norbin affect the subcellular localization of each other, and to quantify their abundance in the cytosol and membrane, respectively, we carried out subcellular fractionation of HEK-293 cells expressing EE-P-Rex1 and/or myc-Norbin, using mock-transfected cells as a control. Total lysates of serum-starved cells were cleared of debris and nuclei by low-speed centrifugation, the postnuclear supernatant fractionated further into membrane and cytosol fractions, and the amounts of P-Rex1 and Norbin protein in each fraction were determined by Western blotting with 6F12 P-Rex1 and Norbin C1 antibody (Fig. 8*A*).

When EE-P-Rex1 was expressed alone, P-Rex1 protein was largely found in the cytosol fraction, as expected (1, 26, 27), and 11% of P-Rex1 from the postnuclear supernatant were recovered in the membrane fraction. In contrast, coexpression with myc-Norbin decreased the amount of P-Rex1 protein in the cytosol fraction and increased its presence in the membrane fraction to 28% (Fig. 8, *A* and *B*). Similarly, when myc-Norbin was expressed alone, Norbin protein was largely found in the cytosol fraction, as expected (38), and 9.5% of Norbin from the postnuclear supernatant was distributed in the membrane fraction. Coexpression with EE-P-Rex1 reduced the amount of Norbin in the cytosol and increased its localization in the membrane fraction to 29% (Fig. 8, *A* and *C*). These data show that P-Rex1 and Norbin stimulate the membrane localization of each other, thereby confirming the results obtained by immunofluorescence microscopy. The Norbin-dependent increase in P-Rex1 membrane localization was comparable in its extent to the previously described membrane translocation of P-Rex1 that is induced synergistically by co-stimulation with Gβγ and PIP_3_ (26). Norbin is therefore a major regulator of the subcellular localization of P-Rex1.

##### Summary of P-Rex1 Regulation by Norbin

**FIGURE 9.**
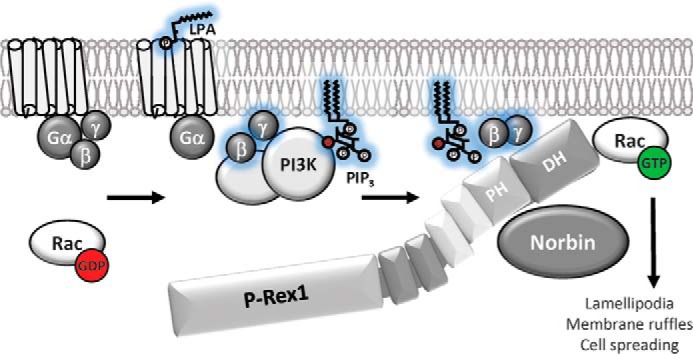
**Model of P-Rex1 regulation by Norbin.** The GPCR adaptor protein Norbin interacts directly with P-Rex1 through the PH domain and stimulates the basal Rac1-GEF activity of P-Rex1. Norbin also proportionally increases the Rac1-GEF activity of P-Rex1 when stimulated through the lipid second messenger PIP_3_ that is generated by PI3K, or through the Gβγ subunits of heterotrimeric G proteins that are released upon GPCR stimulation. In response to cell stimulation with the GPCR ligand LPA, Norbin promotes the P-Rex1-dependent activation of endogenous Rac1. The interaction of Norbin with P-Rex1 also leads to a robust translocation of both proteins from the cytosol to the plasma membrane. Finally, coexpression of P-Rex1 and Norbin induces cell morphologies characteristic of active Rac1, namely lamellipodia formation, membrane ruffling, and cell spreading. We propose that the increased membrane localization of P-Rex1 caused by Norbin binding brings the Rac-GEF into closer contact with its other membrane-bound activators, PIP_3_ and Gβγ, and with its substrate Rac1, thereby promoting Rac1 activity and Rac1-dependent cell responses.

In summary, we have identified herein the GPCR adaptor protein Norbin as a novel regulator of the Rac-GEF P-Rex1. We have shown that Norbin interacts directly with P-Rex1, stimulates the Rac1-GEF activity of P-Rex1 *in vitro* and *in vivo*, induces a robust translocation of P-Rex1 from the cytosol to the plasma membrane, and stimulates P-Rex1 and Rac1-dependent cell morphologies (lamellipodia, membrane ruffling, and cell spreading). Therefore, Norbin regulates P-Rex1 activity, localization, and cellular functions (Fig. 9). We propose that Norbin is a major regulator of the subcellular localization of P-Rex1 (and vice versa) and that, by recruiting P-Rex1 to the plasma membrane, Norbin brings P-Rex1 into closer proximity with its other activators, PIP_3_ and Gβγ, and with its substrate Rac1, thereby facilitating the P-Rex1-mediated activation of Rac1 and Rac1-dependent remodeling of cytoskeletal structure to alter cell morphology.

## Discussion

To activate Rac1, P-Rex1 needs to be localized at the plasma membrane, yet P-Rex1 is largely cytosolic in basal (serum-starved) cells. We and others previously reported that the translocation of P-Rex1 from the cytosol to the plasma membrane is mediated synergistically by Gβγ and PIP_3_, in response to cell stimulation (26, 27). We have found here that Norbin is also largely cytosolic in basal cells, confirming an earlier study (38). Surprisingly, both P-Rex1 and Norbin translocated to plasma membrane when coexpressed, even in basal cells where Gβγ and PIP_3_ levels should be low. The effect of Norbin on the membrane localization of P-Rex1 was as robust as that stimulated synergistically by PIP_3_ and Gβγ (26), suggesting that Norbin is a major regulator of the subcellular localization of P-Rex1.

Three possible mechanisms have been described previously that might control the subcellular localization of Norbin. Norbin binds the lipid second messenger phosphatidic acid, which is produced by phospholipase D1 (45), and although the functional consequences of this interaction remain unknown, it is conceivable that phosphatidic acid might recruit Norbin to the plasma membrane upon cell stimulation. Furthermore, the N terminus of Norbin can be palmitoylated, and this palmitoylation has been shown to recruit Norbin to early endosomes in hippocampal neurons (52). However, the plasma membrane localization of Norbin was not observed in that study, so palmitoylation is unlikely to be the mechanism of the plasma membrane recruitment we describe here. Finally, Norbin interacts directly and constitutively with many GPCRs (41, 43, 44), and also interacts *in vivo* with semaphorin 4C, a transmembrane protein that controls axon path finding and synapse structure (46). It is possible that P-Rex1 binding to Norbin increases the propensity of Norbin to bind to these plasma-membrane-localized proteins, and that P-Rex1 and Norbin are thus retained at the plasma membrane by multi-protein complex formation.

Norbin increased the Rac1-GEF activity of P-Rex1 directly *in vitro*, but its effect was minor compared with P-Rex1 activation by PIP_3_ or Gβγ (1, 18). In addition, the fold-stimulation of P-Rex1 activity by Norbin was similar, regardless of whether PIP_3_ or Gβγ were present, which suggested that Norbin might activate P-Rex1 by increasing its availability of P-Rex1 in the assay. As Norbin promotes the membrane localization of P-Rex1 substantially, and as the effect of Norbin on P-Rex1 Rac1-GEF activity seen *in vivo* was larger than *in vitro*, it is therefore likely that Norbin promotes P-Rex1 Rac1-GEF activity largely by increasing its membrane localization, and that the liposomes used *in vitro* insufficiently reproduce the complex composition of the plasma membrane. In addition, the bacterially produced Norbin used *in vitro* lacks post-translational modifications that might be important for activating P-Rex1 efficiently. Furthermore, P-Rex1 is activated synergistically by Gβγ and PIP_3_, and LPA stimulation leads directly to the release of Gβγ subunits and indirectly to the activation of PI3K (and therefore PIP_3_ production). Norbin may be a stronger activator of P-Rex1 during such synergistic activation, and/or the P-Rex1·Norbin complex may contain additional proteins *in vivo* that contribute. The latter possibility is supported by our finding that a larger N-terminal region of P-Rex1 is required for Norbin to stimulate the membrane translocation of P-Rex1 than for mere Norbin binding. As Norbin binds directly to the cytoplasmic tail of many GPCRs, and as P-Rex1 is directly activated by Gβγ subunits upon GPCR stimulation, it is tempting to speculate that P-Rex1·Norbin complexes with these proteins might exist in the cell.

We have shown by mutagenesis that the PH domain of P-Rex1 is required for Norbin binding. A recent crystal structure of the P-Rex1 DH/PH domains has shown that the PH domain does not contact the DH domain during catalysis (20). Therefore, Norbin binding to the PH domain may not directly impact on the ability of the DH domain to bind and activate Rac1, although this is a possibility. We have previously shown that PIP_3_ binds to and activates P-Rex1 via the PH domain (1, 18), and modeling on the basis of the crystal structure predicted that PIP_3_ may bind on the opposite face of the DH/PH tandem than Rac1 (20). In addition, it was proposed that Gβγ would contact both the DH and PH domains, again on the opposite side to Rac1 (20). Our data showing that Norbin enhances the activation of P-Rex1 by PIP_3_ and Gβγ suggests that Norbin binding to the PH domain occurs in a manner that does not interfere with their binding. Furthermore, as the ΔPDZ mutant of P-Rex1 bound more weakly to Norbin than the full-length protein, the PDZ domain may also contribute to Norbin binding. Structural predictions for Norbin suggest that the protein is almost entirely comprised of α-helices that resemble HEAT repeats (36). By analogy with HEAT-repeat proteins such as importin β1 (54), Norbin is likely to fold into a curved elongated super-helical structure with a concaved surface that confers protein binding through hydrophobic interactions over a relatively large area rather than a specific binding motifs within.

We have demonstrated that coexpression of P-Rex1 and Norbin promotes lamellipodia formation, membrane ruffling, and cell spreading more strongly than P-Rex1 expression alone. P-Rex1 remodels the structure of the actomyosin cytoskeleton through its function as a Rac-GEF. Through this role, P-Rex1 has been shown to control the morphology of several cells types, including endothelial cells, leukocytes, fibroblasts, and neurons, and to affect many processes that depend on cytoskeletal structure, including cell adhesion, migration, secretion, and synaptic plasticity (1, 2, 6, 11). We found that Norbin alone induced a limited degree of membrane ruffling that did not reach statistical significance, unlike its P-Rex1 dependent effects. A role of Norbin in lamellipodia formation and membrane ruffling has not been described before but is not surprising, considering that its first reported function was its ability to promote neurite outgrowth in Neuro-2a cells (33). Similarly, Norbin was first isolated on the basis of being up-regulated in rat hippocampus upon chemical induction of long-term potentiation (33), and was later shown to be an important regulator of synaptic plasticity in mice (41). Both neurite outgrowth and synaptic plasticity require Rac1 activity, and similar cytoskeletal dynamics as those generating lamellipodia and membrane ruffles (51). Previous studies have attempted to address the mechanism through which Norbin might promote neurite outgrowth. Mutagenesis showed that an N-terminal fragment of Norbin confers neurite outgrowth more strongly than the full-length protein, suggesting the possibility of autoinhibition (47), although through which mechanism such autoinhibition might be relieved is unknown. In addition, Norbin was shown to bind Dia1 (47), a RhoA-target protein that mediates actin filament nucleation and microtubule stabilization (55). However, as Norbin affected neither the actin polymerizing nor microtubule stabilizing activities of Dia1 (47), it seems unlikely that Dia1 is a major mediator of Norbin-induced neurite outgrowth. Therefore, the mechanism through which Norbin regulates neurite outgrowth and synaptic plasticity remained elusive. Our results showing that Norbin enhances P-Rex1 function by stimulating its plasma membrane localization and Rac1-GEF activity and that Norbin induces robust lamellipodia, ruffles, and spreading in cells that express P-Rex1 provide such a possible mechanism.

We have shown that the tissue distribution of Norbin is more widespread than previously appreciated, which suggests that the interaction between P-Rex1 and Norbin may affect Rac1-dependent responses in many cell types that express both proteins, including neutrophils, where P-Rex1 is known to have important functions in the inflammatory response (1, 2, 4–6, 9). It will therefore be important in the future to assess the impact of the P-Rex1/Norbin interaction on the physiology of the whole organism, through combined genetic deficiency in mice. Finally, P-Rex1 has an important pathophysiological role in cancer progression and metastasis, with up-regulation of P-Rex1 protein being particularly common in breast cancer and melanoma (2, 13, 17). In addition, a recent report showed that P-Rex1 levels are down-regulated in autistic individuals from the Chinese Han population, and that P-Rex1 deficiency in mice causes autism-like social behavior (53). Our results showing that Norbin levels in some areas of the mouse brain are affected by P-Rex1 deficiency suggested that their expression levels may be linked. It might therefore be valuable in the future to manipulate Norbin levels in mouse models of mammary tumor growth (17) and melanoma metastasis (13), to assess whether Norbin alters the pathophysiological roles of P-Rex1 in these cancers.

In conclusion, we have shown here that the Rac-GEF P-Rex1 and the GPCR-adaptor protein Norbin are binding partners and major regulators of the subcellular localization of each other. By recruiting P-Rex1 to the plasma membrane, Norbin brings P-Rex1 into close proximity with its other activators and its substrate Rac1, thereby facilitating the P-Rex1-dependent activation of Rac1 and Rac1-dependent cell responses such as changes in cell morphology. It seems likely that Norbin will also control other P-Rex1 Rac-GEF functions, such as adhesion, migration, and ROS formation. Inversely, it is plausible that the known functions of Norbin in neuronal morphology and plasticity that underlie its protective role in depression and schizophrenia (41, 42) are mediated in large part through its interaction with P-Rex1.

## Author Contributions

D. P., M. A. B., K. H., M. J. B., J. M. T., D. O., and H. C. E. W. designed, performed, and analyzed experiments. D. P., M. A. B., and H. C. E. W. wrote the manuscript.

## Supplementary Material

Supplemental Data

## Footnotes

3 The abbreviations used are:

GEF: guanine-nucleotide exchange factor
DEP: Dvl, EGL-10, and pleckstrin homology
DH: Dbl homology
eGFP: enhanced GFP
Gβγ: Gβγ subunits of heterotrimeric G proteins
GPCR: G-protein coupled receptor
GTPγS: guanosine 5′-3-*O*-(thio)-trisphosphate
IP4P: inositol polyphosphate 4-phosphatase
LPA: lysophosphatidic acid
mGluR5: metabotropic glutamate receptor-5
PAE: pig aortic endothelial
PH: pleckstrin homology
P-Rex1: PIP_3_-dependent Rac exchanger 1
PIP_3_: phosphatidylinositol (3,4,5)-trisphosphate
PP1α: protein phosphatase 1α
SIM: structured illumination microscopy.
