# Supplementary material for: Norbin Stimulates the Catalytic Activity and Plasma Membrane Localization of the Guanine-Nucleotide Exchange Factor P-Rex1
Source: J Biol Chem. 2016 Jan 20;291(12):6359–75. doi: 10.1074/jbc.M115.686592 (PMC4813545; doi:10.1074/jbc.M115.686592)
Supplement: Supplemental Data [file supp_291_12_6359__index.html]

Norbin Stimulates the Catalytic Activity and Plasma Membrane Localization of the Guanine-Nucleotide Exchange Factor P-Rex1 — P-Rex1 Regulation by Norbin — Supplemental Data 

# Norbin Stimulates the Catalytic Activity and Plasma Membrane Localization of the Guanine-Nucleotide Exchange Factor P-Rex1

## Supplemental Data

**Files in this Data Supplement:**

- Supplemental Movie Legend
- Supplemental Movie S1
