# Supplementary material for: Norbin Stimulates the Catalytic Activity and Plasma Membrane Localization of the Guanine-Nucleotide Exchange Factor P-Rex1
Source: J Biol Chem. 2016 Jan 20;291(12):6359–75. doi: 10.1074/jbc.M115.686592 (PMC4813545; doi:10.1074/jbc.M115.686592)
Supplement: Supplemental Data [file supp_M115.686592_legend_for_Movie_1.docx]

**Movie 1. Colocalization of P-Rex1 and Norbin in PAE cell membrane ruffles.** 3D stack of super-resolution SIM images of the PAE cell shown in Figure 6B that expresses eGFP-P-Rex1 (green) and myc-Norbin (red). SIM images were acquired using a Nikon dual mode SIM/STORM super resolution microscope, and Nikon Elements software was used to create the 3D stack from 30 planes of 0.12 μm depth.
